# Questioning the Role of *BRAF* V600E as a Prognostic Indicator in Papillary Thyroid Carcinoma

**DOI:** 10.1245/s10434-025-16963-0

**Published:** 2025-02-05

**Authors:** Mahsa Javid

**Affiliations:** University of Louisville, Louisville, KY USA

The question of whether a *BRAF* V600E mutation portends a poorer prognosis in papillary thyroid cancer (PTC) has long been asked of this most prevalent mutation in PTC. For more than 2 decades, there have been conflicting reports as to its association with worse patient outcomes. Even the American Thyroid Association hedged their opinion by incorporating *BRAF* V600E into their risk stratification system without recommending its routine evaluation.^1^ There is no longer any clear evidence that the mutation truly correlates with high-risk PTC.

In their recent study, Lai et al.^2^ not only reinforce the questionability of *BRAF* V600E mutation as a predictive tool, but also report that its absence is associated with more aggressive disease in PTC. There are several factors that can clarify the reason for the strikingly contrasting findings in this and multiple other studies that showed the mutation has no association with prognostic factors such as lymph node metastasis, extrathyroidal extension, multicentricity, recurrence, and long-term outcome, and prior studies that asserted that such was the case. Primarily, the retrospective nature of the studies connotes the bias that they are subject to and that they cannot account for confounding factors. In many cases, it is challenging, if at all possible, to ascertain the differences in study populations that lead to divergent results—such factors are relegated to descriptions such as ‘unknown, unrelated reasons’. In this instance though, there is an apparent distinction in the V600E wild-type populations in the different studies, which may explain to some degree the contradicting results alluded to by Lai et al.^2^: the inclusion of benign lesions, now classified as noninvasive follicular thyroid neoplasm with papillary-like nuclear features (NIFTP), in the PTC groups in reports prior to the 2017 WHO classification change. It is plausible that misleading conclusions were drawn about an association between the V600E mutation and more aggressive disease when such benign (and V600E wild-type) lesions were included in the cancer groups. The degree to which this classification change affected the statistical results depends on the prevalence of NIFTP and cannot be entirely determined without re-analyzing the prior studies using the updated designations. A recent cohort study regarding the effect of the 2017 WHO classification reported a significant 8% increase in the incidence of *BRAF* V600E mutations in PTC since the categorization of NIFTP was introduced.^3^ Interestingly, the previous studies that demonstrated a relationship between V600-positive tumors and aggressive features of PTC reported an overall mutation prevalence of under 50%, whereas the mutation prevalence was much higher (70–80%) in studies that did not establish such a relationship. The prevalence of *RAS*-mutated follicular variant PTCs in the different populations may explain to some degree the differing study findings.

A logical inference to draw from less contested data, i.e. that the V600E mutation is highly prevalent whereas the occurrence of poor outcomes such as recurrence and disease-specific mortality (10% and 5%, respectively) is rare, is that there is no link between the mutation and these adverse events. Importantly, a relationship with the V600E mutation and distant metastatic disease, which is arguably one of the most important determinants of outcome, has not been found.^4^

Several observations on the molecular level may elucidate the reasons for this actuality: (1) that it is not just the presence of *BRAF* V600E mutation that affects PTC phenotype but rather the percentage of V600E-mutant alleles,^5,6^ data that are not usually collected in outcomes-based studies; (2) that *BRAF* V600E PTC does not consist of a single homogenous group but represents a diverse group of tumors with a least four molecular subtypes with varying levels of differentiation;^7^ and (3) that co-mutations of V600E with others such as with the *TERT* promotor are associated with high-risk clinicopathological characteristics. These discoveries augment the concept that the V600E mutation cannot be used as a sole determinant of aggressive PTC.

Selection bias and other limitations are apparent in both the study by Lai et al.^2^ and other studies, including the introduction of routine mutational analysis only part way through the studies, testing only for V600 and few or no other mutations, and changes in the analysis and DNA extraction methods during the studies, which affect sensitivity of detection. Differing diagnostic, management, and surveillance protocols also influence the extent to which recurrence is identified.

As much as it is desirable to have a tumor marker, particularly one that can be identified preoperatively, to act as a prognostic indicator, such that management decisions can be based on its presence or absence, our current understanding of the molecular complexity of PTC indicates that *BRAF* V600E cannot be used as an independent predictor of prognosis. There is a need to further investigate both the V600 wild-type and mutation-positive subpopulations for indicators of higher risk. New discoveries will have mushrooming prognostic and therapeutic implications, the likes of which we are fortunate enough to be able to imagine.
